# Magnetic Alignment of Electrospun Fiber Segments Within a Hydrogel Composite Guides Cell Spreading and Migration Phenotype Switching

**DOI:** 10.3389/fbioe.2021.679165

**Published:** 2021-06-16

**Authors:** Harrison L. Hiraki, Daniel L. Matera, Michael J. Rose, Robert N. Kent, Connor W. Todd, Mark E. Stout, Anya E. Wank, Maria C. Schiavone, Samuel J. DePalma, Alexander A. Zarouk, Brendon M. Baker

**Affiliations:** ^1^Department of Biomedical Engineering, University of Michigan, Ann Arbor, MI, United States; ^2^Department of Chemical Engineering, University of Michigan, Ann Arbor, MI, United States; ^3^Department of Mechanical Engineering, University of Michigan, Ann Arbor, MI, United States

**Keywords:** electrospinning, fiber alignment, hydrogel, epithelial cell migration, tendon

## Abstract

Fibrous extracellular matrix (ECM) proteins provide mechanical structure and adhesive scaffolding to resident cells within stromal tissues. Aligned ECM fibers play an important role in directing morphogenetic processes, supporting mechanical loads, and facilitating cell migration. Various methods have been developed to align matrix fibers in purified biopolymer hydrogels, such as type I collagen, including flow-induced alignment, uniaxial tensile deformation, and magnetic particles. However, purified biopolymers have limited orthogonal tunability of biophysical cues including stiffness, fiber density, and fiber alignment. Here, we generate synthetic, cell-adhesive fiber segments of the same length-scale as stromal fibrous proteins through electrospinning. Superparamagnetic iron oxide nanoparticles (SPIONs) embedded in synthetic fiber segments enable magnetic field induced alignment of fibers within an amorphous bulk hydrogel. We find that SPION density and magnetic field strength jointly influence fiber alignment and identify conditions to control the degree of alignment. Tuning fiber length allowed the alignment of dense fibrous hydrogel composites without fiber entanglement or regional variation in the degree of alignment. Functionalization of fiber segments with cell adhesive peptides induced tendon fibroblasts to adopt a uniaxial morphology akin to within native tendon. Furthermore, we demonstrate the utility of this hydrogel composite to direct multicellular migration from MCF10A spheroids and find that fiber alignment prompts invading multicellular strands to separate into disconnected single cells and multicellular clusters. These magnetic fiber segments can be readily incorporated into other natural and synthetic hydrogels and aligned with inexpensive and easily accessible rare earth magnets, without the need for specialized equipment. 3D hydrogel composites where stiffness/crosslinking, fiber density, and fiber alignment can be orthogonally tuned may provide insights into morphogenetic and pathogenic processes that involve matrix fiber alignment and can enable systematic investigation of the individual contribution of each biophysical cue to cell behavior.

## Introduction

Stromal extracellular matrix (ECM) provides manifold biophysical cues that direct both physiologic and pathologic cell behavior. A major component of stromal ECM are fibrous proteins (e.g., collagens, fibronectin, and elastin) that serve as cell-adhesive scaffolding and provide structural and mechanical support to a variety of tissues (Poltavets et al., 2018). Cells dynamically deposit, reorganize, and respond to the fibrous architecture of the ECM (Dallas et al., 2006). Through contact guidance cues, fibrous protein structures direct a variety of morphogenetic processes including tenogenesis, branching morphogenesis, and angiogenesis (Kirkpatrick et al., 2007; Brownfield et al., 2013; Iannone et al., 2015). Fibrous proteins are also heavily implicated in initiating and directing invasion from primary tumors during breast cancer progression (Provenzano et al., 2006). Second harmonic generation (SHG) imaging has provided valuable insights into collagen architecture during morphogenesis and disease progression (Ingman et al., 2006). With this insight, biomaterials recapitulating aligned fibrous architectures have been developed to model and direct such processes *in vitro*.

Purified biopolymers such as type I collagen and fibrin have been used to model stromal ECM as both possess fibrous topography. However, polymerization of these materials under typical conditions produces hydrogels with isotropic fibrous architecture due to the stochastic nature of fibrillogenesis (Wakuda et al., 2018). To better model highly anisotropic fibrous architecture, such as that found in tendons and around primary breast tumors, several approaches to align collagen fibers have been developed. Early methods to align collagen gels took advantage of the slight negative charge of collagen to align fibers with powerful magnetic fields (Dickinson et al., 1994). More recently, a diversity of methods to align collagen gels have emerged including flow-induced alignment (Han et al., 2016), embedding of magnetic particles which are dragged through the gel with an external magnetic field (Guo and Kaufman, 2007; Provenzano et al., 2008; Carey et al., 2016; Taufalele et al., 2019), application of tensile forces via stretching (Riching et al., 2015), and fibroblast-mediated reorganization of fibers (Dumont et al., 2013; Ray et al., 2017). These methods create highly anisotropic collagen gels and have been instrumental in investigating how aligned fibrous architecture influences cell behavior. However, purified biopolymers typically have limited orthogonal control of relevant biophysical cues (Wolf et al., 2009; Mason et al., 2013). For example, increasing type I collagen gel concentration leads to commensurate increases in fiber density, stiffness, and ligand density. In contrast, synthetic hydrogels (e.g., polyethylene glycol, methacrylated gelatin, and functionalized polysaccharides) offer enhanced orthogonal tunability of these physical properties. However, these amorphous hydrogels typically lack fibrous architecture (Baker and Chen, 2012; Li et al., 2017).

Electrospinning offers a means to generate fibrous topography that closely recapitulates the geometry and length-scale of fibrous proteins found in stromal ECM. The electrospinning process uses a voltage gradient to draw solid fibers from a charged polymer solution. Previous work with PVA, PLGA, and dextran methacrylate has shown that cell migration on electrospun, synthetic fiber matrices captures key aspects of cell migration in fibrous natural ECM proteins like type I collagen (Kim et al., 2016; Wang et al., 2018; Hong et al., 2019). Recent work from our group and others have established means to generate embedded fiber segments within amorphous synthetic hydrogels (Rose et al., 2017, 2018; Matera et al., 2019). These hydrogel composites enable cell studies within topographically complex fibrous environments in which fiber density and stiffness can be orthogonally controlled. However, such composites rely on encapsulating fiber segments within a hydrogel, resulting in a random distribution of embedded fibers.

In this work, we embedded superparamagnetic iron oxide nanoparticles (SPIONs) within our synthetic fiber segments during the electrospinning process to enable fiber alignment under an externally applied magnet field. Degree of fiber alignment within a 3D amorphous hydrogel proved sensitive to SPION density as well as the strength of the imposed magnetic field. Computational modeling of the magnetic field revealed dependence upon magnet placement, where hydrogels appropriately positioned during crosslinking can achieve homogeneous alignment of constituent fibers. We find that fiber length influences the frequency of entanglement as a function of fiber density during magnetic alignment. Finally, we demonstrate the use of magnetic fiber alignment within our hydrogel composite system to control encapsulated tendon fibroblast (tenocyte) alignment and elicit directional migration of a breast epithelial cell line. Interestingly, we find that fiber alignment not only biases epithelial cell migration direction, but also promotes cell-cell breakage events leading to a switch in 3D migration phenotype.

## Materials and Methods

### Reagents

All reagents were purchased from Sigma Aldrich and used as received, unless otherwise stated.

### Synthesis of Modified Dextran Vinyl Sulfone

#### Dextran Vinyl Sulfone (DVS)

Dextran was functionalized with vinyl sulfone pendant groups using a previously described protocol (Matera et al., 2019). Briefly, linear high molecular weight dextran (MW 86,000 Da; MP Biomedicals) was reacted with pure divinyl sulfone (Fisher) under basic conditions (pH 13.0). Functionalization was terminated through pH adjustment to 5.0 with hydrochloric acid. Reaction products were dialyzed against milli-Q water for 3 days, with water changed twice daily. Purified products were then lyophilized for 3 days and reconstituted at 100 mg mL^–1^ in a Michael-type addition buffer (MTAB; 1 N NaOH, 1 M HEPES, 1 mg mL^–1^ phenol red in milli-Q water).

### DVS Fiber Segment Fabrication

DVS was dissolved at 0.6 g mL^–1^ in a 1:1 mixture of milli-Q water and dimethylformamide. SPIONs with or without polyvinylpyrrolidone (PVP) coating (US Research Nanomaterials) were added at 2.5, 5, or 10 w/v%. Lithium phenyl-2,4,6-trimethylbenzoylphosphinate (LAP) photoinitiator (10 v/v%) and methacrylated rhodamine (2.5 v/v%) (Polysciences, Inc.) were added to the solution to facilitate photoinitiated crosslinking and fluorescent visualization, respectively. Polymer solutions were electrospun in a humidity-controlled glove box held at 21°C and 30–35% relative humidity. Electrospinning was performed at 0.25 mL hr^–1^ flow rate, 7 cm gap distance, and −9.0 kV voltage onto a grounded copper collective surface. Fibers were collected on glass cover slides and crosslinked under ultraviolet light (100 mW cm^–2^) for 20 sec. Custom fabricated chrome photomasks were placed over fiber mats during UV photocrosslinking to control fiber length. Fiber mats were detached from cover slides into milli-Q water and broken into individual fiber segments. Fiber segments were purified through a series of centrifugation steps to remove uncrosslinked polymer and entangled fibers before resuspension in MTAB at 10 v/v%. Prior to encapsulation within bulk hydrogels, fibers were coupled with 2.0 mM RGD (CGRGDS; CPC Scientific) via Michael-type addition to enable eventual cell adhesion.

### Magnetic Gelation Chamber Fabrication

The magnet housing apparatus was created with solid 1/2′′ aluminum and 1.00′′× 2.00′′ T-slotted aluminum. Two N52 neodymium magnets (K&J Magnetics) were housed in carriages made from solid aluminum, securing the magnets on either side with a hole removed at the face of the magnet. The carriages were attached to the T-slotted aluminum rails on the base of the setup, which aligned the carriages and allowed them to controllably slide along the rails with a crankshaft. A 3D printed clamp was designed to hold a petri dish containing fiber-reinforced hydrogel composites within the center of the magnet axis. To prevent encapsulated fibers and cells from settling during hydrogel crosslinking, an Arduino-controlled stepper motor with a 3D-printed grip was programmed to flip the petri dish 180° every 20 s.

### Computation Visualization of Magnet Field Lines

COMSOL Multiphysics software was used to quantify magnetic flux densities and visualize field lines between magnets. A 3D stationary study modeling magnetic fields generated without current (permanent magnet) was performed. Two cylinders (1.905 × 3.81 cm) modeling the two magnets were placed within a 15–25 cm radius sphere to model air impedance. Surface flux density of the two cylinders was set to 661.9 mT, the innate surface field of N52 neodymium magnets. A single slice in the X-Z plane was generated to visualize magnetic flux density around and between the magnets. White arrows were overlaid to visualize magnetic field lines with the arrow size logarithmically proportional to strength of magnet flux along the field line. To generate 1D plots of magnetic flux density in the Z-direction across various magnet spacings, flux density values were extracted from a path along the magnet axis.

### Hydrogel Formation and Fiber Alignment

DVS gels were formed via an analogous click reaction at 3.1 w/v% with 9.64 mM VPMS cross-linker and heparin binding peptide (2 mM). All hydrogel precursor solutions were made in PBS. To create fibrous hydrogels, a defined stock solution (10 v/v%) of suspended fiber segments in MTAB was mixed into hydrogel precursor solutions prior to gelation. Via controlling the dilution of the fiber suspension, fiber density was tuned at a constant hydrogel weight percentage and bulk stiffness. Hydrogel precursor solutions were injected into 5 mm in diameter polydimethylsiloxane gaskets and crosslinked at 37°C for 1 h. To align encapsulated fibers, hydrogels were crosslinked between the two magnets of magnetic gelation chamber.

### Cell Lines and Culture

#### Primary Tenocyte Harvesting

Mouse resident tenocytes were harvested with a previously established protocol (Shimada et al., 2014). Briefly, mouse tail tendons were encapsulated in 2 mg mL^–1^ collagen I (Advanced Biomatrix), allowing cells to proliferate into the gel for 11 days. Gels were then digested with 0.25 mg mL^–1^ collagenase from *C. histolyticum* and cells were centrifuged out. Tenocytes were cultured in DMEM supplemented with 10 v/v% fetal bovine serum (Fisher) and 1 v/v% penicillin/streptomycin/amphotericin B. Tenocytes were passaged near confluency at a 1:2 ratio and used for studies until passage 3. For 3D hydrogel encapsulation studies, media was additionally supplemented with 50 ng mL^–1^ L-ascorbic acid-2-phosphate and transforming growth factor-β3 (Peprotech). Human mammary epithelial cells MCF10A (ATCC) were cultured in DMEM/F12 (1:1) supplemented with 5 v/v% horse serum (Fisher), 20 ng mL^–1^ rhEGF (Peprotech), 0.5 mg mL^–1^ hydrocortisone, 100 ng mL^–1^ cholera toxin, and 10 μg mL^–1^ insulin (Fisher). MCF10As were passaged at confluency at a 1:4 ratio and used for studies until passage 8. *Spheroid formation*: MCF10As were detached with 0.25% trypsin-EDTA (Life Technologies), counted, and formed into 200 cell-sized spheroids overnight in inverse pyramidal PDMS microwells (AggreWell^TM^, Stem Cell Technologies) treated with 0.5% Pluronic F-127 to prevent cell adhesion. All cells were cultured at 37°C and 5% CO_2_.

### Cytotoxicity Screens

SPIONs with or without PVP coating were suspended in complete MCF10A media over a range of densities. To create SPION conditioned media, SPIONs were incubated in complete MCF10A media for 48 h and then centrifuged out at 20,000 rcf for 30 min. *2D monolayer assay:* MCF10A cells plated on glass coverslips were exposed to SPION containing media or SPION conditioned media for 12 h then incubated in serum free MCF10A media with Hoechst stain (1 μg/mL) and propidium iodide (1 μg/mL) for 20 min prior to fixing. *3D hydrogel assay:* DVS fiber segments containing SPIONs with or without PVP coating were coencapsulated with single MCF10A cells (1,000,000 cells mL^–1^) in DVS hydrogels. After 12 h in culture, hydrogels were incubated in serum free MCF10A media with Hoechst stain (2 μg/mL) and propidium iodide (2 μg/mL) and incubated on a rocker plate at 0.33 Hz for 1 h to enhance diffusive transport prior to fixing.

### Single Cell Spreading Studies

Primary-derived tenocytes (5,000,000 cells mL^–1^) and fiber segments (3 v/v%) were co-encapsulated in DVS hydrogels. Studies were maintained in complete tenocyte media for 7 days, with media replenished every other day.

### Spheroid Migration Studies

MCF10A spheroids were harvested and centrifuged to remove residual single cells. Spheroids (6 000 mL^−1^) and fiber segments (3 v/v%) were co-encapsulated in DVS hydrogels. Studies were cultured in complete MCF10A media for 6 days, with media replenished every other day.

### Fluorescence, Staining, and Microscopy

Samples were fixed with 4% paraformaldehyde for 1 h at room temperature. To visualize the actin cytoskeleton and nuclei, samples were stained with phalloidin and DAPI for 1 h at room temperature. For immunostaining, gels were additionally permeabilized in PBS containing Triton X-100 (5 v/v%), sucrose (10 w/v%), and magnesium chloride (0.6 w/v%) and blocked in 4% BSA. Fluorescent imaging was performed with a Zeiss LSM 800 laser scanning confocal microscope. For migration analysis, Z-stacks were acquired with a 10x objective. All images are presented as maximum intensity projections.

### Cell Migration Analysis

A previously established custom MATLAB image analysis code was used to extract morphometric data from spheroid migration studies (Hiraki et al., 2021). Briefly, max intensity projections of spheroid nuclei and F-actin channels were separately thresholded and object size filtered to remove background. A user-drawn ellipsoidal ROI covering the spheroid body was used to separate the spheroid body from migratory cells within outgrowths. The code segmented F-actin structures into individual outgrowths which were defined as either contiguous or non-contiguous based on contiguity with the spheroid body. A separate function segmented overlapping nuclei to identify all nuclei within outgrowths. Individual outgrowth F-actin masks were used to determine migration distance into the surrounding hydrogel utilizing a separate custom function. Corresponding individual nuclei masks were used to determine nuclei locations, nuclear counts, and mark non-contiguous outgrowths as either multicellular clusters or single cells. All individual outgrowth nuclei and F-actin masks were then summed to produce final images of nuclei and F-actin channels. Individual outgrowth nuclei and F-actin masks were saved with counted nuclei or plotted lengths, respectively, and assigned an index to address discrepancies or outliers within final quantified data. Resulting data were stratified by contiguity with the spheroid body and exported to a spreadsheet containing individual outgrowth indices, number of migratory cells, outgrowth areas, and migration distances. Finally, spheroid body and outgrowth masks were summed across all analyzed spheroids to produce heatmap overlays.

### Statistics

Statistical significance was determined by one-way analysis of variance (ANOVA) with *post hoc* analysis (Tukey test), with significance indicated by *p* < 0.05. All data are presented as mean ± standard deviation.

## Results

### Fabrication of a Magnetically Responsive Electrospun Fiber Segments

**FIGURE 1:**
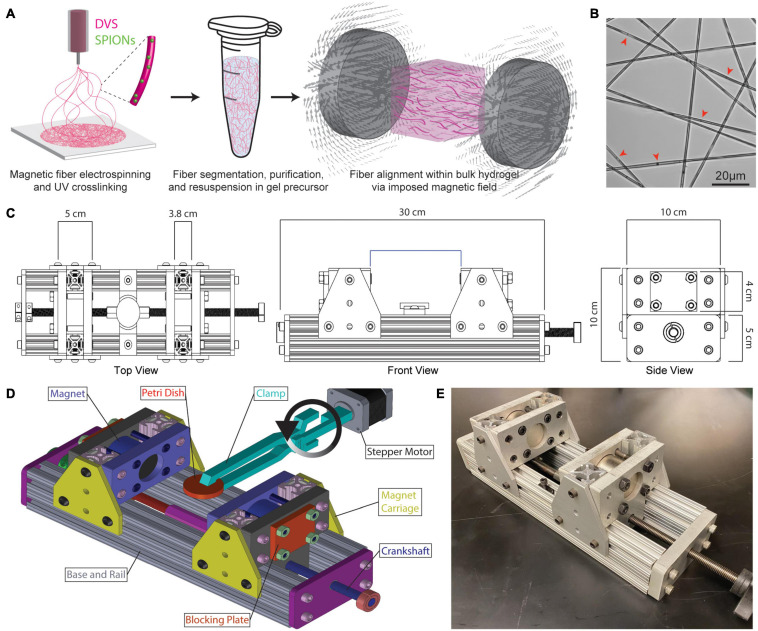
Fabrication and magnetic alignment of SPION-containing electrospun DVS fiber segments. **(A)** Schematic overview of DVS polymer electrospinning, collected fiber suspension within a bulk hydrogel precursor solution, and alignment of SPION-containing fibers within an externally applied magnetic field. **(B)** Transmitted light image of SPIONs within electrospun DVS fibers. Red arrowheads indicate SPIONs. **(C)** Top, front, and side views of magnetic gelation chamber containing variably spaced neodymium magnets to control magnetic field strength during hydrogel gelation. **(D)** AutoCAD rendering of the magnetic gelation chamber with Arduino-controlled stepper motor to flip hydrogel composites during crosslinking and prevent fiber settling. **(E)** Photo of the final magnetic gelation chamber.

To create fiber segments on the same length-scale as fibrous proteins found in stromal ECM, DVS polymer solution was electrospun to produce fibers ∼2 μm in diameter (Matera et al., 2019). Electrospun fiber mats were processed into suspensions of fiber segments which could then be encapsulated in 3D hydrogels and aligned by an externally applied magnetic field (Figure 1A). SPIONs added to the electrospinning solution were stably encapsulated within fibers upon photocrosslinking (Figure 1B, red arrowheads). To define the strength of an imposed magnetic field during hydrogel gelation, a magnetic gelation chamber was designed with adjustable spacing of two N52 neodymium permanent magnets (Figure 1C). The setup consisted of an aluminum base and rails upon which two magnet carriages housing the neodymium magnets could be controllably spaced with a crankshaft over a range of 6–20 cm. A hydrogel precursor solution containing DVS fiber segments was crosslinked within a petri dish positioned between the two magnets. To prevent fibers or cells from settling during hydrogel crosslinking, a clamp attached to an Arduino-controlled stepper motor flipped the petri dish 180° within the magnetic field every 20 s during gelation (Figure 1D). This setup enabled facile control over fiber alignment via magnet spacing and resulting magnetic field strengths (Figure 1E).

### Computational Visualization of Magnetic Field Lines and Field Strength

**FIGURE 2:**
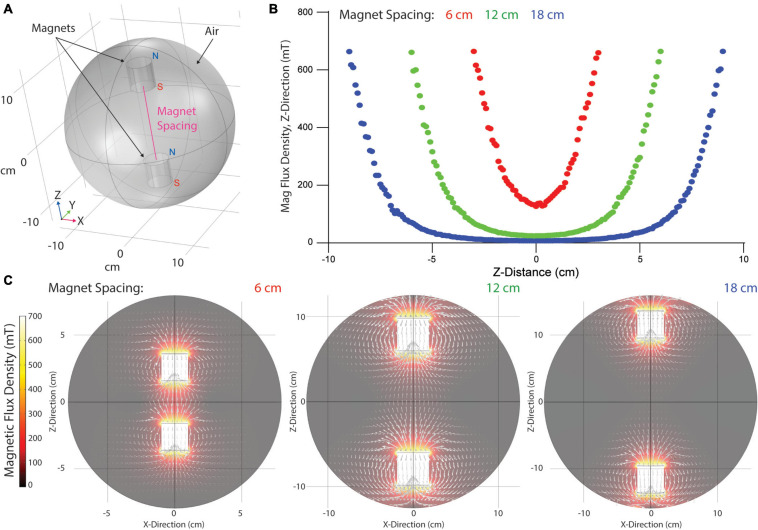
Computational modeling of applied magnetic fields within gelation chamber. **(A)** Model geometry with two cylindrical magnets within a spherical air area. Magnet orientation with North in the positive Z-direction. **(B)** Quantified magnetic field strength in the Z-direction of the magnet axis over a range of magnet spacings. **(C)** Visualization of magnetic flux density and field lines (white arrows).

Magnetic field lines produced by a single permanent magnet resemble concentric ellipses radiating from the magnet’s north to south pole. When opposite poles of two juxtaposed permanent magnets are aligned, field lines combine and densify as a function of spacing between the magnets. To determine the strength of the magnetic field produced within the magnetic gelation chamber, we modeled magnetic flux density using COMSOL. A 3D model of two permanent magnets was created by placing two cylinders of equivalent geometry within a sphere to model air impedance (Figure 2A). Surface fields of 669.1 mT were set at the cylinder surfaces to model N52 neodymium boundary conditions. Magnet flux density along the major magnet axis (Z-axis) was determined across a range of magnet spacings to quantitate the magnetic field strength applied to centrally positioned hydrogels (Figure 2B). Flux density along the Z-axis was parabolic in strength, highest at the magnet surfaces and decaying exponentially to the center position between magnets (Z = 0 cm). The smallest magnet spacing achievable (6 cm) produced a flux density of 126.9 mT at the center. Increasing magnet spacing to 12 and 18 cm significantly decreased flux density to 24.8 and 7.5 mT, respectively. To better visualize field lines between magnets, flux density heat maps were generated and overlaid with white arrows logarithmically proportional to regional flux densities (Figure 2C). Field lines were parallel to magnet axis orientations and decayed exponentially once outside of the radius the magnets (X < −1.905 cm or X > 1.905 cm). Thus, hydrogel composites positioned within the central region of the magnets are exposed to a nearly homogeneous magnetic field with field lines running parallel to the magnets’ axes.

### Degree of Fiber Alignment Is Jointly Regulated by SPION Density and Magnet Spacing

**FIGURE 3:**
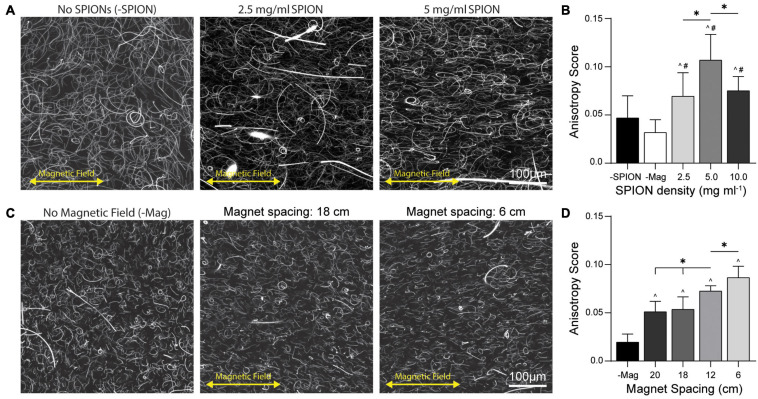
Fiber alignment as a function of SPION density and magnet spacing. **(A)** Fiber alignment at 1 v/v% fiber density in 3D DVS hydrogels across a range of encapsulated SPION densities at 6 cm magnet spacing. **(B)** FibrilTool quantification of anisotropic fiber alignment. **(C)** Fiber alignment of 5 mg mL^–1^ SPION fibers at 1 v/v% over a range of magnet spacings and **(D)** quantification of fiber alignment. All data presented as mean ± standard deviation (SD); ^∗^indicates a statistically significant comparison with *p* < 0.05; ^ indicates significance against –Mag; # indicates significance against –SPION.

To optimize DVS fiber alignment, we first modulated the density at which SPIONs were incorporated into the DVS electrospinning solution. We noted a slight decrease in fiber segment yield with increasing SPION density (data not shown), likely due to the SPIONs interfering with the electrospinning process. Fiber segments were encapsulated in 3D DVS gels at 1 v/v% and aligned at a magnet spacing of 6 cm (Figure 3A). Degree of fiber alignment was quantified via anisotropy score generated with the ImageJ plugin FibrilTool (Boudaoud et al., 2014). Hydrogels containing fibers without SPIONs crosslinked at 6 cm magnet spacing resulted in randomly oriented fibers (Figure 3B; -SPION), indicating DVS fiber segments are not innately responsive to a magnetic field. Hydrogels containing fibers with the highest SPION density (10 mg mL^–1^) crosslinked outside of the magnetic gelation chamber also resulted in randomly oriented fibers (Figure 3B; -Mag), indicating SPION-containing fibers do not align in the absence of an external magnetic field. In contrast, hydrogels containing SPION fibers crosslinked within the magnetic field contained aligned fibers oriented in the direction of the magnetic field. The highest degree of fiber alignment resulted from a SPION density of 5 mg mL^–1^, suggesting a density of 2.5 mg mL^–1^ was below an optimal density required for magnetic forces to align fibers. Conversely, at 10 mg mL^–1^, SPIONs began to form large aggregates within the electrospinning solution, potentially decreasing the total amount retained in fiber segments and therefore limiting alignment (Gutiérrez et al., 2019). We next assessed alignment of 5 mg mL^–1^ SPION-containing fibers across a range of magnetic field strengths by varying the spacing between the two magnets (Figure 3C). A step-wise decrease in fiber alignment was observed with increasing magnet spacing (Figure 3D), indicating fiber segments can be aligned with field strengths between 5 and 125 mT (Figure 2) and that the degree of alignment is a function of both SPION density and field strength. To further visualize degree of fiber alignment, we utilized OrientationJ to produce color map images based on fiber orientation for fibers aligned across SPION encapsulation densities and magnet distances (Supplementary Figure 1; Püspöki et al., 2016).

### Decreasing Fiber Length Prevents Entanglement at High Fiber Density

**FIGURE 4:**
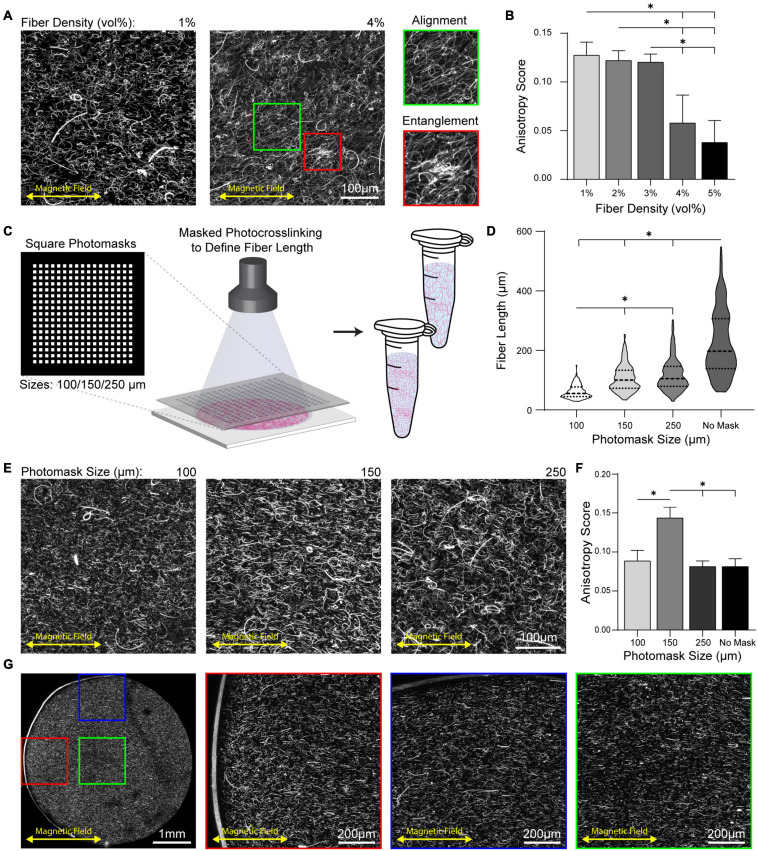
Decreasing fiber length prevents entanglement at high fiber encapsulation density. **(A)** Alignment of full length fibers across a range of densities. Inserts show local regions of alignment (green) and entanglement (red). **(B)** Anisotropy scoring across a range of fiber densities. **(C)** Schematic of photomasking during photocrosslinking of fiber mats to define shorter fiber lengths. **(D)** Quantification of fiber length as a function of photomask size. **(E)** Alignment of fiber segments produced with photomasks within 3D hydrogel at 5 v/v% fiber density and **(F)** corresponding anisotropy scores. **(G)** Cross-section of a 5 mm cylindrical hydrogel composite with fibers produced by 150 μm photomask aligned by 6 cm magnet spacing. Inserts show location regions of fiber aligned at boundaries perpendicular (red) and parallel (blue) to fiber alignment and within the gel center (green). All data presented as mean ± SD; ^∗^ indicates a statistically significant comparison with *p* < 0.05.

Previous reports on approaches to align type I collagen gels have noted collagen fiber entanglement (Ng and Swartz, 2003, 2006; Guo and Kaufman, 2007; Omidinia-Anarkoli et al., 2017). As a high fiber density is key to modeling fibrous tissues such as tendons and the stroma of breast tissue during cancer progression (Provenzano et al., 2006; Docheva et al., 2015), we next determined if increases in fiber density led to entanglement. Fiber density was modulated through the input fiber volume fraction of the hydrogel precursor solution over a range of 1–5 v/v% and gels were crosslinked at a magnet spacing of 6 cm. At fiber densities at or below 3 v/v%, highly anisotropic fiber alignment was achieved with minimal evidence of entanglement (Figures 4A,B). However, at 4 v/v% fiber density, entanglement was apparent which led to a significant decrease in fiber alignment (Figure 4B). Within these gels, heterogeneously distributed regions of localized fiber alignment vs. entanglement were observed (Figure 4A, green and red inserts). At 5 v/v% fiber density nearly all fibers were entangled in large clumps, leading to an anisotropy score similar to non-aligned fibers (Figure 3). As fiber entanglement occurred in regions where long fiber segments were co-encapsulated in high proximity, we sought to maintain alignment at higher fiber densities by decreasing fiber segment length. To do so chrome photomasks were placed over electrospun fiber mats during photocrosslinking (Figure 4C). Photomasks with arrays of square patterns (100, 150, or 250 μm) yielded fiber segments spanning 60–120 μm in average length; in contrast, fibers generated without photomasking were on average 225 μm in length with considerably larger variance (Figure 4D). To test if shorter fibers diminished entanglement despite high encapsulation density, 5 v/v% fibrous hydrogels were crosslinked at 6 cm magnet spacing. Fibers created with the 100 and 150 μm photomasks were highly aligned and showed little evidence of entanglement, while gels containing 250 μm photomasked fibers possessed regions of entanglement similar to fibers generated without photomasking (Figure 4E). Despite the lack of evident entanglement in either hydrogel, gels containing 150 μm photomasked fibers had a significantly higher anisotropy score compared to gels containing 100 μm photomasked fibers. This difference is likely due to the influence of object length in FibrilTool’s calculation of anisotropy score (Figure 4F). To determine if rigid boundaries locally influenced fiber alignment, we imaged a cross-section of a 5 mm cylindrical hydrogel composite containing 150 μm photomasked fibers. No regional differences in fiber alignment at gel boundaries perpendicular or parallel to fiber alignment were observed, indicating that magnetic alignment overcame any flow-induced alignment along boundaries (Figure 4G). In sum, magnetic alignment of 5 mg mL^–1^ SPION-containing fibers optimally sized by photomasking resulted in homogeneous alignment.

### SPION Encapsulation Within Fiber Segments Prevents Cytotoxic Interaction With Cells

**FIGURE 5:**
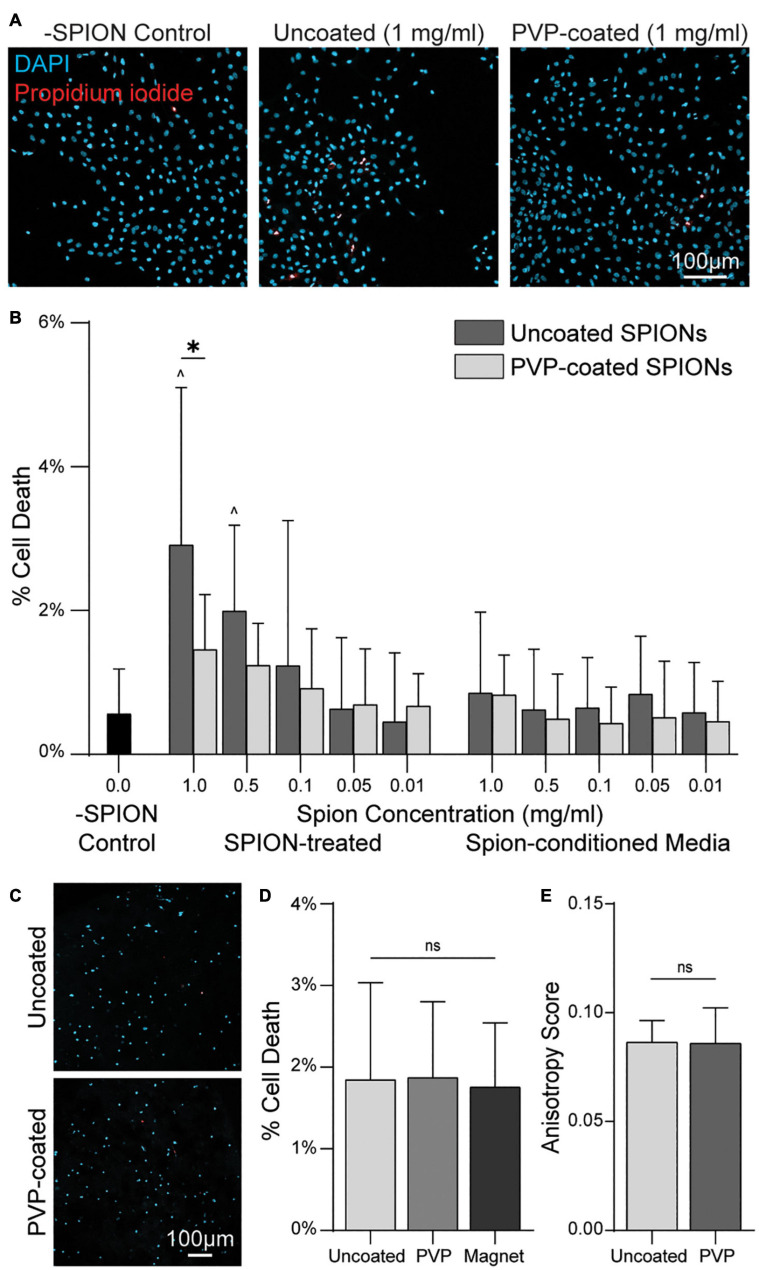
PVP-coated SPIONs improve cytocompatibility without compromising magnetic alignment. **(A)** Hoechst and propidium iodide (PI) staining of MCF10As with uncoated or PVP-coated SPIONs added to culture media for 12 h. **(B)** Quantification of MCF10A death as measured by % PI^+^ nuclei with either SPIONs directly added to media (SPION-treated) or SPIONs incubated in media and then removed prior to media transfer to cells (SPION-conditioned Media). **(C)** Hoechst/PI staining of single MCF10As encapsulated alongside SPION fibers in DVS hydrogels after 12 h of culture. Non-fibrous gel exposed to a magnetic field (Magnet). **(D)** Corresponding quantification of % cell death. **(E)** Alignment of fibers containing SPIONs with or without PVP coating. All data presented as mean ± SD; ^∗^indicates a statistically significant comparison with *p* < 0.05; ^ indicates significance against no SPION control.

The presence of charged SPIONs has previously been reported to be cytotoxic (Rose et al., 2017; Patil et al., 2018). To determine if the SPIONs used here are cytotoxic, and to test if cytotoxicity results from direct interaction of SPIONs with cells versus changes in media ion concentrations due to the addition of SPIONs, we added SPIONs or SPION-conditioned media to MCF10A mammary epithelial cell monolayers. Cell death, assessed via staining with membrane-impermeable propidium iodide (PI), was SPION dose-dependent with 1 and 0.5 mg mL^–1^ SPION concentration in media resulting in significant increases in cell death relative to controls (Figures 5A,B). SPION-conditioned media at any concentration tested did not increase cell death above control levels, suggesting cytotoxicity resulted from direct cell interactions with SPIONs rather than changes in media ion concentrations. Polyvinylpyrrolidone (PVP) coating of biomaterials has previously been reported to reduce cytotoxicity (Zhi et al., 2013). As such, we repeated cytotoxicity experiments with PVP-coated SPIONs. A PVP-coated SPION dose-dependent increase in cell death was again observed, but cell death upon addition of 1 mg mL^–1^ SPIONs was lower with PVP coating. PVP-coated SPION-conditioned media did not induce cell death above control levels (Figure 5B). The significant decrease in cell death at the highest SPION concentration indicates PVP coating decreases cytotoxicity. However, it is worth noting the degree of cell death is minimal (< 3%) regardless of PVP coating. To assess cytotoxicity when SPIONs are embedded within fiber segments, we next co-encapsulated single MCF10A cells and fibers containing 5 mg mL^–1^ of SPIONs with or without PVP coating (Figure 5C). After 12 h in culture, no difference in cell death was observed, indicating insignificant SPION escape from fiber segments and limited cytotoxicity. Furthermore, no increase in cell death was observed in non-fibrous gels exposed to the strongest magnetic field (6 cm magnet spacing), indicating cells are not negatively affected by an externally applied magnetic field (Figure 5D). The ability to electrospin SPION-containing fibers or align fibers within 3D gels was not altered by PVP coating (Figure 5E), and therefore PVP-coated SPIONs were used in all subsequent studies.

### Fiber Alignment Directs Uniaxial Spreading in Primary Derived Mouse Tenocytes

**FIGURE 6:**
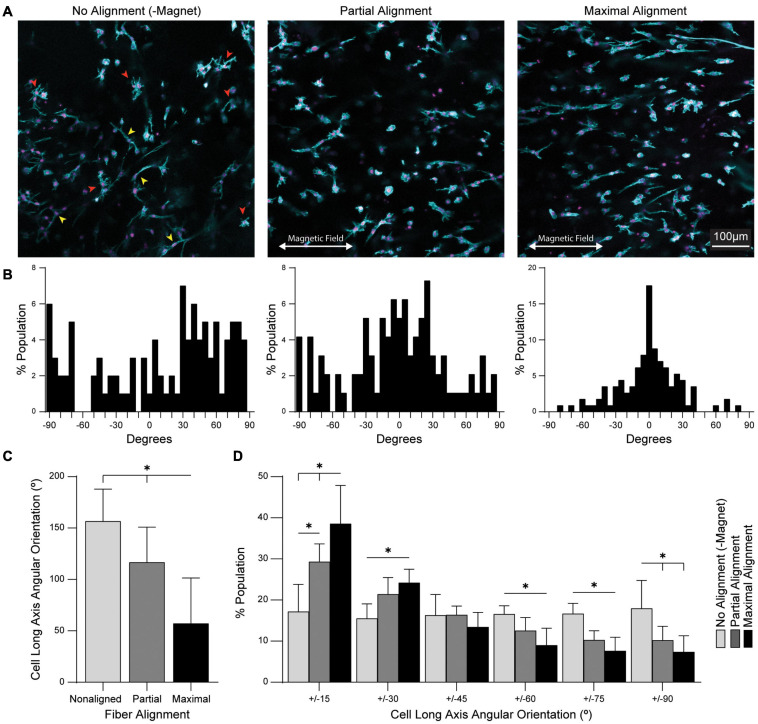
Fiber alignment directs the orientation and morphology of encapsulated tendon fibroblasts. **(A)** Fluorescent images of primary mouse tendon fibroblasts (tenocytes) cultured in hydrogel composites for 7 days. Red arrowheads indicate stellate morphology cells; yellow arrowheads indicate uniaxially spread cells. **(B)** Histograms of cell orientation as a function of fiber alignment. **(C)** Full width-half max quantification of n = 10 cell orientation distributions. **(D)** Angular stratification of cell orientations as a function of fiber alignment. All data presented as mean ± SD; ^∗^indicates a statistically significant comparison with *p* < 0.05.

Alignment of fibrous ECM architecture is known to influence fibroblast spreading and polarization. For applications in tendon tissue engineering, alignment of tendon fibroblasts (tenocytes) within 3D hydrogels may be critical to mechanosensing and ECM deposition (Schoenenberger et al., 2018; Xu et al., 2021). To enable fibroblast adhesion to magnetic fibers, residual VS groups were functionalized with the cell-adhesive peptide, CGRGDS, via Michael-type addition. Primary tenocytes harvested from mouse tendons were co-encapsulated along with SPION-containing fibers in a bulk MMP-degradable DVS hydrogel to determine the influence of fiber alignment on tenocyte spreading and orientation. Magnet spacing was modulated to produce aligned (6 cm), partially aligned (12 cm), or non-aligned (no magnetic field) hydrogel composites. Tenocyte spreading in non-aligned gels included both stellate morphologies in which filopodia extended in all directions (Figure 6A, red arrowheads) and uniaxial spread morphologies with high aspect ratios (Figure 6A, yellow arrowheads). In contrast, tenocyte spreading in both partially aligned and aligned gels favored higher aspect ratios with the long axes of cells oriented in the direction of fiber alignment. Fiber alignment at the highest possible field strength (6 cm magnet spacing) resulted in significantly more aligned cells than lower field strength (12 cm magnet spacing), as calculated by the full width half max of cell orientation distributions (Figures 6B,C). Quantification of individual cell orientations revealed nearly random distributions in non-aligned gels. In partially aligned and aligned gels, the distribution of cell orientation increasingly narrowed with the majority of cells oriented within ± 30 degrees of the fiber alignment axis (Figure 6D).

### Fiber Alignment Directs Multicellular Migration and Induces Migration Phenotype Switching

**FIGURE 7:**
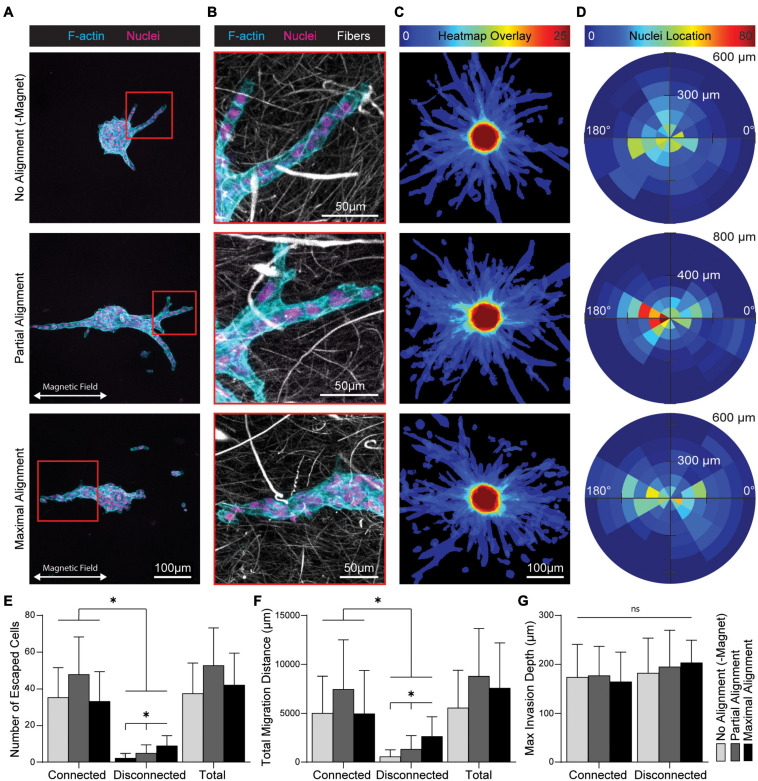
Fiber alignment biases migration direction from multicellular MCF10A spheroids and induces cell-cell breakage events. **(A)** Fluorescent images of cell outgrowth from multicellular MCF10A spheroids encapsulated in DVS hydrogel composites after 6 days. **(B)** Higher magnification image including DVS fibers from location depicted by inset in **(A)**. **(C)** Heatmap overlays created by an aggregate sum of binarized actin channels and **(D)** rose plots of migratory cell nuclei location for *n* = 25 spheroids per condition. Quantification of **(E)** total number of migratory cells and **(F)** total migration distance stratified by outgrowth contiguity with the spheroid. **(G)** Maximum invasion depth of individual outgrowths stratified by contiguity with the spheroid. All data presented as mean ± SD; ^∗^indicates a statistically significant comparison with *p* < 0.05.

ECM fiber alignment has also been heavily implicated in epithelial cell migration during transtromal escape from primary tumors. To examine the effect of fiber alignment on epithelial cell migration, MCF10A spheroids and SPION-containing fibers were co-encapsulated within MMP-degradable DVS gels. Degree of fiber alignment was again modulated by magnetic field strength. Migration from spheroids occurred predominantly as multicellular collective strands that contact guided along fiber segments biased in the direction of fiber alignment (Figure 7A and Supplementary Figure 2). Within partially aligned and aligned fibrous matrices, nuclei also appeared elongated in the direction of fiber alignment (Figure 7B). To more directly visualize migration directional bias, we utilized a previously developed custom MATLAB image analysis code to generate heatmap overlays of actin structures (Figure 7C) and rose plots of nuclear locations (Figure 7D) for 25 spheroids (Hiraki et al., 2021). Non-aligned gels promoted radially uniform cell outgrowths and distribution of nuclei. In contrast, the majority of migratory outgrowths in partially aligned and maximally aligned gels occurred within ± 30 degrees of the axis of fiber alignment. We did not observe a change in the total number of migrating cells across each gel condition, suggesting that fiber alignment does not increase the frequency of cell migration. However, in aligned gels there was an increase in the number of cells migrating as single cells or multicellular clusters disconnected from the main body of the spheroid (Figure 7E). The image analysis code also quantifies total migration distance (the summed migration distance of each cell from the spheroid periphery as a measure of net transtromal migration) and maximum invasion depth (the maximal depth into the surrounding stromal matrix of an outgrowth). Collective strands contiguous to the spheroid accounted for the majority of total transtromal migration distance with no significant change as a function of fiber alignment. However, we noted a significant increase in total migration distance of disconnected migratory cells at both levels of fiber alignment (Figure 7F). Despite the emergence of distinct migratory phenotypes, no change in maximum invasion distance was observed across different degrees of fiber alignment or between connected or disconnected phenotypes (Figure 7G). In sum, these data suggest fiber alignment does not increase overall cell migration or migration speed but rather increases directional migration via contact guidance and the frequency of cell-cell breakage events that engender disconnected invasive cell structures.

## Discussion

Here, we describe a means to align magnetic electrospun fibers within a 3D hydrogel composite that models stromal ECM. SPIONs were stably incorporated into DVS fiber segments, enabling control over the density and alignment of fibrous architecture via an externally applied magnetic field. SPION density and magnetic field strength jointly contributed to fiber alignment, enabling fine control over fiber alignment. Fiber entanglement due to the length and density of fiber segments impaired alignment, but shortening fibers via photomasking prevented fiber entanglement during magnetic alignment, thereby increasing the range of achievable fiber densities in 3D hydrogel composites. We found that both the spreading of individually encapsulated cells and orientation of multicellular migratory structures from spheroids were influenced by the degree of fiber alignment. Aligned fibrous architecture directed uniaxial spreading of primary derived mouse tenocytes in lieu of stellate morphologies. Fiber alignment also biased the direction of multicellular migration from MCF10A spheroids and increased the number of cell-cell breakage events, leading to the emergence of invading single cells and multicellular clusters. While previous methods have been developed to aligned fibers within purified biopolymer hydrogel such as type I collagen, the synthetic fiber-reinforced hydrogel composite system presented here provides more facile orthogonal tuning of fibrous architecture parameters including the degree of alignment, fiber length, and fiber density.

Our custom designed magnetic gelation chamber holds two small N52 neodymium magnets that produce a surface field of 661.9 mT. In comparison to previous methods utilizing Tesla-range magnetic fields to align type I collagen gels (Dickinson et al., 1994), alignment of SPION-containing DVS fiber segments requires a significantly lower magnetic field strength achievable with small rare earth magnets without the need for specialized or expensive equipment (Figure 2). In comparison to other methods of aligning fibers, such as flow-induced alignment or fibroblast-mediated matrix reorganization, control over magnetic fiber fabrication and field strength provide a higher degree of control of fiber alignment. As anticipated, we found fiber alignment to be sensitive to both SPION density within fiber segments and field strength (Figure 3). As such, we were able to tune the degree of fiber alignment within hydrogel composites to produce different degrees of alignment. Enhanced control over the degree of alignment could enable modeling of progressive stages of tissue repair or pathogenesis that involve matrix fibers such as tendon regeneration or invasive ductal carcinomas, respectively (Provenzano et al., 2006; Schoenenberger et al., 2018). Furthermore, the degree of fiber alignment could be tuned to reflect histologic samples or *in situ* images of tissue to more accurately model specific tissue types or states of disease.

As the stroma possesses a high density of fibrous ECM proteins, we next modulated fiber density within our hydrogel composites and observed fiber entanglement and reduced alignment when the density of fibers exceeded 3 v/v%. To prevent entanglement, we used chrome photomasks to shorten fibers (Figure 4). Photomasking decreased variance in fiber lengths and enabled increased alignment at higher fiber densities. The large variance in fiber length without photomasking is likely due to the processing of deposited fibers mats into individual fiber segments, which involves vortexing resuspended fiber mats—an uncontrolled process yielding fibers between 100 and 550 μm in length. In contrast, photomasking produced more consistent fiber lengths. Alignment of shorter fiber segments did not result in entanglement and proved insensitive to boundary effects (Figure 4G). Fibers near the glass coverslip bottom and sides of the PDMS gasket were aligned to the same degree as fibers within the center of the gel. In comparison to flow-induced fiber alignment, which creates alignment artifacts near rigid boundaries, magnetic alignment readily overcomes initial fiber orientation resulting from the injection of hydrogel precursor solution.

Functionalization of fiber segments with cell-adhesive RGD allowed cells to engage and spread along fiber segments and respond to matrix alignment. We encapsulated primary derived mouse tenocytes into aligned hydrogel composites and observed cell spreading along fiber segments and a morphologic transition from stellate to uniaxial morphologies oriented in the direction of fiber alignment (Figure 6). Alignment of tenocytes has potential implications in tendon wound repair, as the cells and matrix within this tissue are highly organized (Docheva et al., 2015). Given the non-contact nature of this method, alignment of cell-laden hydrogels within in vivo wound sites is an exciting possibility currently under exploration.

Similar to single tenocyte spreading, we observed multicellular migration from MCF10A spheroids biased in the direction of fiber alignment (Figure 7). Cells migrating as collective strands contact guided along fiber segments with nuclei elongated in the direction of fiber alignment. Interestingly, we noted a significant increase in disconnected migratory outgrowths including single cell and multicellular clusters with fiber alignment. This switch in migratory phenotype may indicate directional migration along aligned matrix fibers promotes EMT signaling and/or decreased cell-cell adhesion to induce cell-cell breakage events (Pearson, 2019). Another explanation is that aligned matrices increase migration speed, causing leading cells to lose adhesion to slower moving trailing cells. Enhanced cell migration speed along aligned fibrous matrices has been reported in 2D settings (Wang et al., 2018; Xue et al., 2018). While we did not observe an increase in net invasion depth with fiber alignment, instantaneous migration speeds were not assessed here. As the bulk DVS hydrogel stiffness/crosslinking is separately defined from fiber density and alignment (Matera et al., 2019; Hiraki et al., 2021), this hydrogel composite can be used to investigate the individual contributions of fiber alignment and hydrogel stiffness/crosslinking on 3D cell migration speed in future studies (Riching et al., 2015; Wang et al., 2018). Further investigation with timelapse imaging could directly assess if fiber alignment increases migration speed during proteolysis-dependent 3D cell migration. Orthogonal tuning of fiber density and alignment at a constant hydrogel stiffness could also provide insight into the influence of tumor-associated collagen signatures (TACS), as previously described by Provenzano et al. (2008). TACS describes three major changes in collagen architecture surrounding solid tumors during breast cancer progression that facilitate metastatic invasion, two of which are increased fiber density and radial alignment of fibers at the tumor-stroma interface. By varying input volume fraction of fiber segments and magnetic field strength, matrix fiber density and alignment can be differentially tuned to model progressive states of tumor stroma.

While RGD was used to enable cell adhesion to fiber segments here, other ECM peptides can be used to model full length proteins such as the GXOGER sequence of type I collagen (DePalma et al., 2021). As DVS fiber segments are not hydrolytically or proteolytically degradable, cell force-mediated reorganization of fibrous architecture could also be investigated. The magnetic, electrospun fiber segments developed here can be easily integrated within other natural and synthetic biomaterials. MMP-cleavable DVS hydrogel was selected as the bulk material here due to its tunability of bulk stiffness and crosslinking via Michael-type addition. For integration with other hydrogels, crosslinking kinetics should be carefully taken into account. Fiber segments were immobilized after 8 min of DVS hydrogel crosslinking via Michael-type addition. The post-gelation degree of fiber alignment is likely a function of magnetic field strength in conjunction with hydrogel precursor solution viscosity as a function of crosslinking. As such, stronger magnets may be required to achieve the same degree of fiber alignment if hydrogel crosslinking kinetics are significantly faster than the DVS hydrogels employed here.

We present a hydrogel composite system consisting of SPION-containing electrospun fiber segments which can be aligned within an externally applied magnetic field. We demonstrate orthogonal tunability of key fibrous matrix attributes including fiber length, fiber density, and degree of fiber alignment. The ability to align magnetic fibers proved insensitive to boundary conditions, allowing homogeneous fiber alignment throughout a millimeter-scale hydrogel. With this system, we demonstrate the ability to align single encapsulated primary mouse tenocytes, which may have utility as an injectable biomaterial therapy to mediate tendon repair. Furthermore, we show control over directional multicellular migration from MCF10A spheroids and find that fiber alignment induces breakage events leading to migration phenotype switching from collective strands to single cells and multicellular clusters. The tunability of fibrous architecture within this hydrogel composite and the ability to integrate magnetic fibers with other biomaterials could enable modeling of stromal tissue architectures for future studies of connective tissue repair and disease processes.

## Data Availability Statement

The raw data supporting the conclusions of this article will be made available by the authors, without undue reservation.

## Author Contributions

HH: first author generating data for fiber alignment, COMSOL modeling, and cell studies. DM: fiber segment fabrication protocol. MR: hydrogel composite fabrication. RK: tenocyte harvesting. CT, MES, AW, and MCS: magnetic gelation chamber fabrication. SD: photomask design and fabrication. AZ: data collection and Fibril Tool quantifications. All authors contributed to the article and approved the submitted version.

## Conflict of Interest

The authors declare that the research was conducted in the absence of any commercial or financial relationships that could be construed as a potential conflict of interest.

## Supplementary Material

Supplementary Figure 1 Degree of fiber alignment is determined by both encapsulated SPION density and magnetic field strength. Color maps produced by OrientationJ of fiber alignment at 1 v/v% fiber density in 3D DVS hydrogels across **(A)** a range of encapsulated SPION densities and **(B)** across a range of magnet spacings.

Supplementary Figure 2 Fiber alignment induces directional migration from MCF10A spheroids. Fluorescent images of cell outgrowth from MCF10A spheroids encapsulated in DVS hydrogel composites after 6 days for various degrees of fiber alignment.
