# Supplementary figures and images for: Magnetic Alignment of Electrospun Fiber Segments Within a Hydrogel Composite Guides Cell Spreading and Migration Phenotype Switching
Source: Front Bioeng Biotechnol. 2021 Jun 16;9:679165. doi: 10.3389/fbioe.2021.679165 (PMC8242362; doi:10.3389/fbioe.2021.679165)

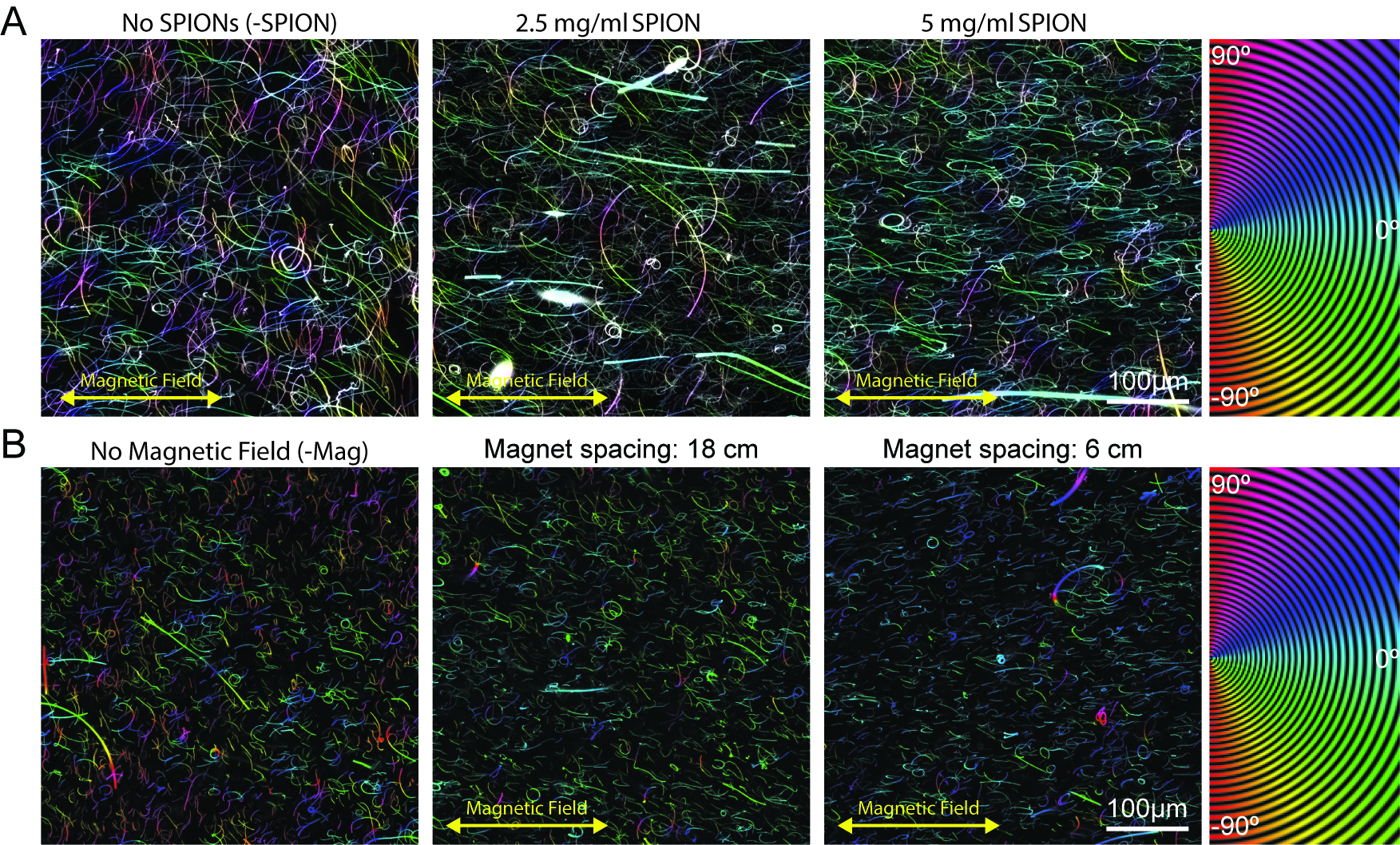

Supplement: Supplementary Figure 1 — Degree of fiber alignment is determined by both encapsulated SPION density and magnetic field strength. Color maps produced by OrientationJ of fiber alignment at 1 v/v% fiber density in 3D DVS hydrogels across (A) a range of encapsulated SPION densities and (B) across a range of magnet spacings. [file Image_1.JPEG]

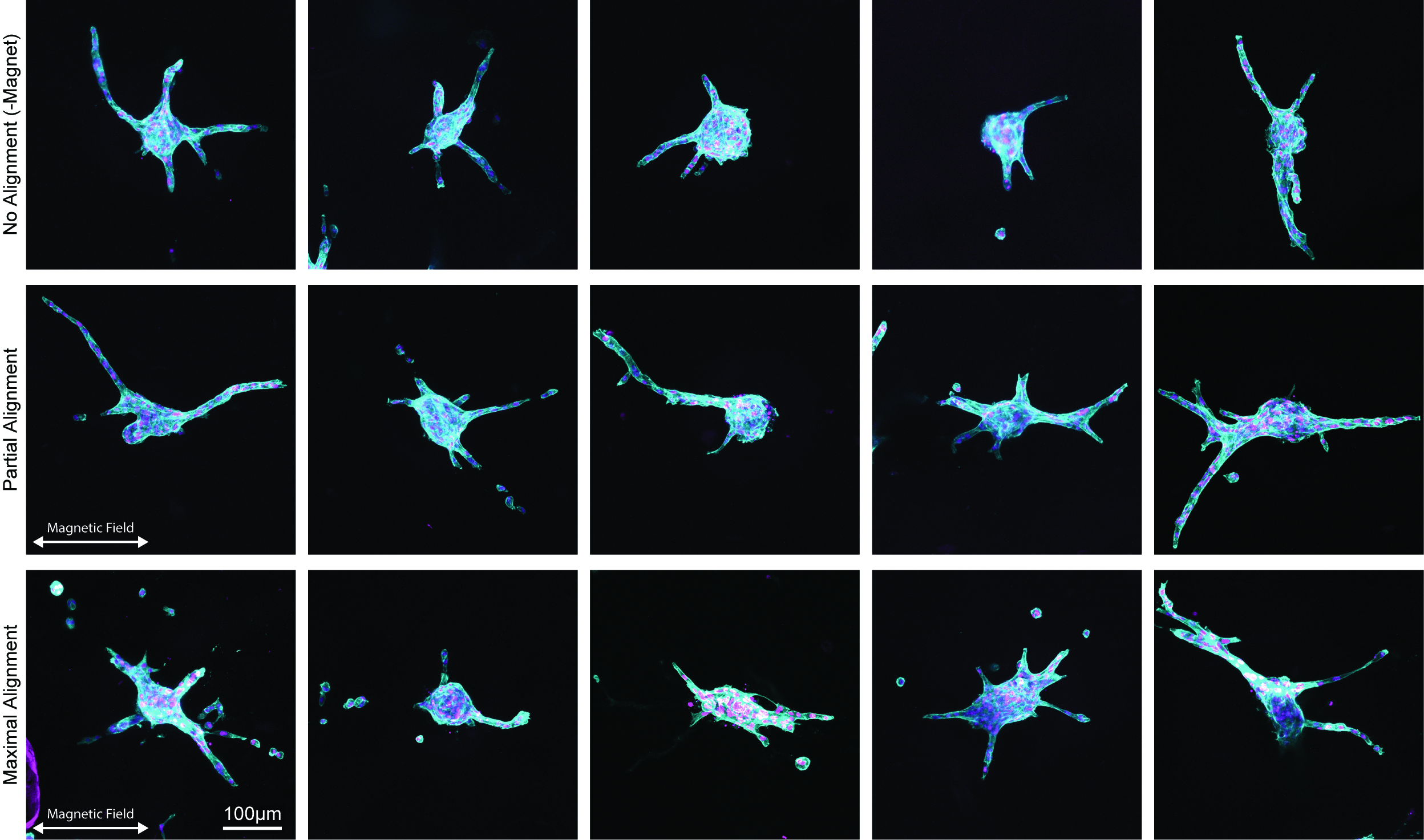

Supplement: Supplementary Figure 2 — Fiber alignment induces directional migration from MCF10A spheroids. Fluorescent images of cell outgrowth from MCF10A spheroids encapsulated in DVS hydrogel composites after 6 days for various degrees of fiber alignment. [file Image_2.jpg]
